# Supplementary material for: Computer-Assisted Design of Environmentally Friendly and Light-Stable Fluorescent Dyes for Textile Applications
Source: Int J Mol Sci. 2019 Nov 27;20(23):5971. doi: 10.3390/ijms20235971 (PMC6928968; doi:10.3390/ijms20235971)
Supplement: Supplementary file 1 [file ijms-20-05971-s001.pdf]

Computer-assisted design of environmentally friendly and light-stable fluorescent dyes for textile applications

Songsong Tang[a], Guoqiang Chen[a*], Gang Sun[b*]

[a]National Engineering Laboratory for Modern Silk, College of Textile and Clothing Engineering, Soochow University, Suzhou, 215123, China

[b]Division of Textiles and Clothing, University of California, Davis, California 95616, United States.

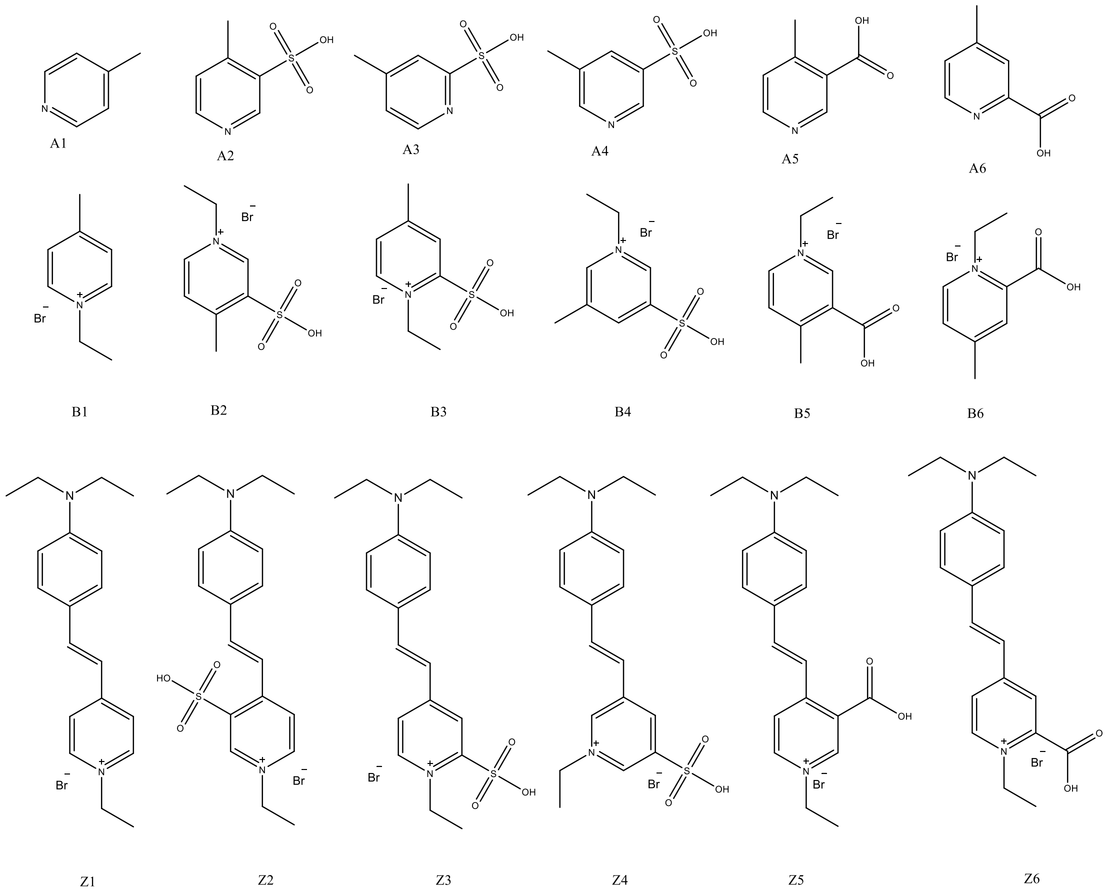

Figure S1. Structures of raw materials (A1, A2, A3, A4, A5, and A6), intermediates (B1, B2, B3, B4, B5, and B6) and dyes (Z1, Z2, Z3, Z4. Z5, and Z6)

* Corresponding authors:
Guoqiang Chen: chenguojiang@suda.edu.cn, College of Textile and Clothing Engineering, Soochow University, Suzhou, 215123, China

Gang Sun: gysun@ucdavis.edu, Division of Textile and Clothing, University of California, Davis, CA 95616, USA.

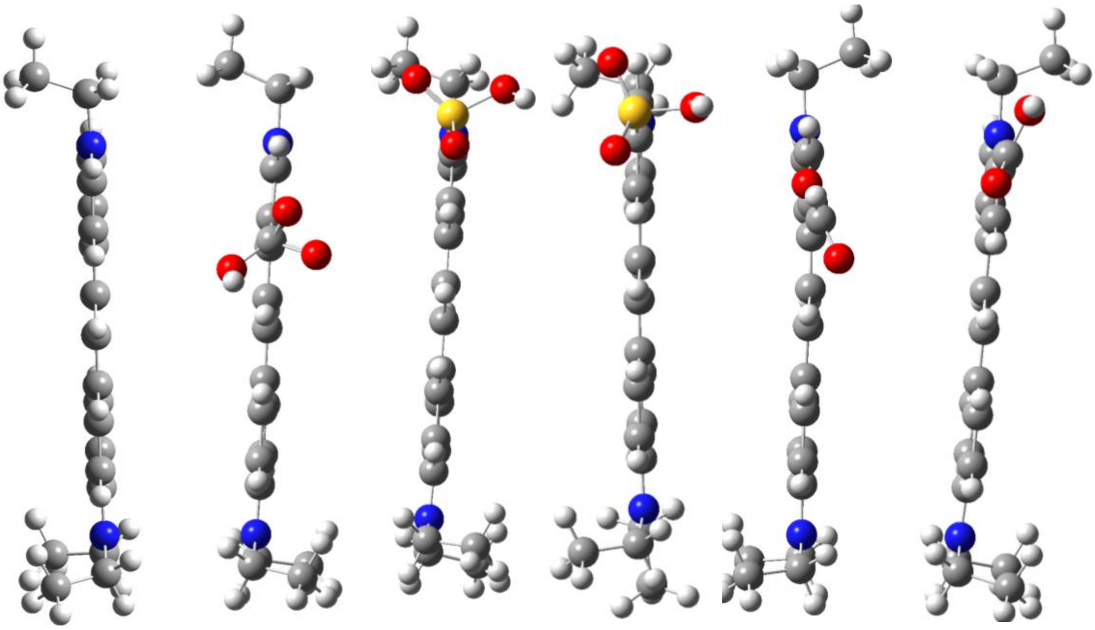

(Z1) (Z2) (Z3) (Z4) (Z5) (Z6)

Figure S2. Geometry structures of the fluorescent dyes (Z1, Z2. Z3, Z4, Z5 and Z6)

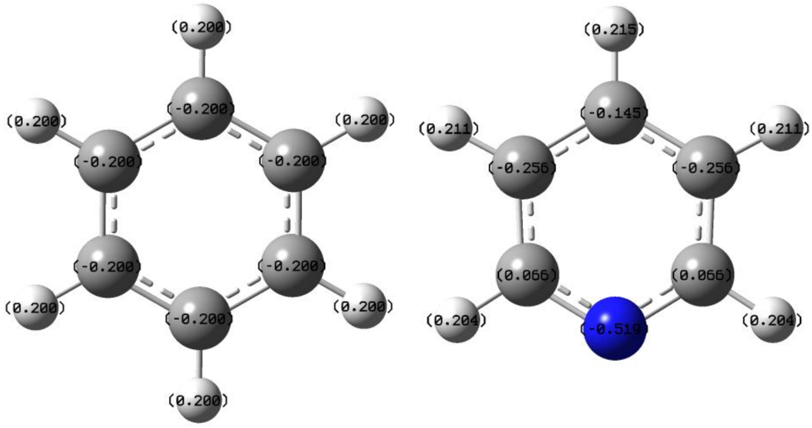

(a) (b)

Figure S3. Charge distributions of pyridine and benzene. (a): benzene, (b): pyridine

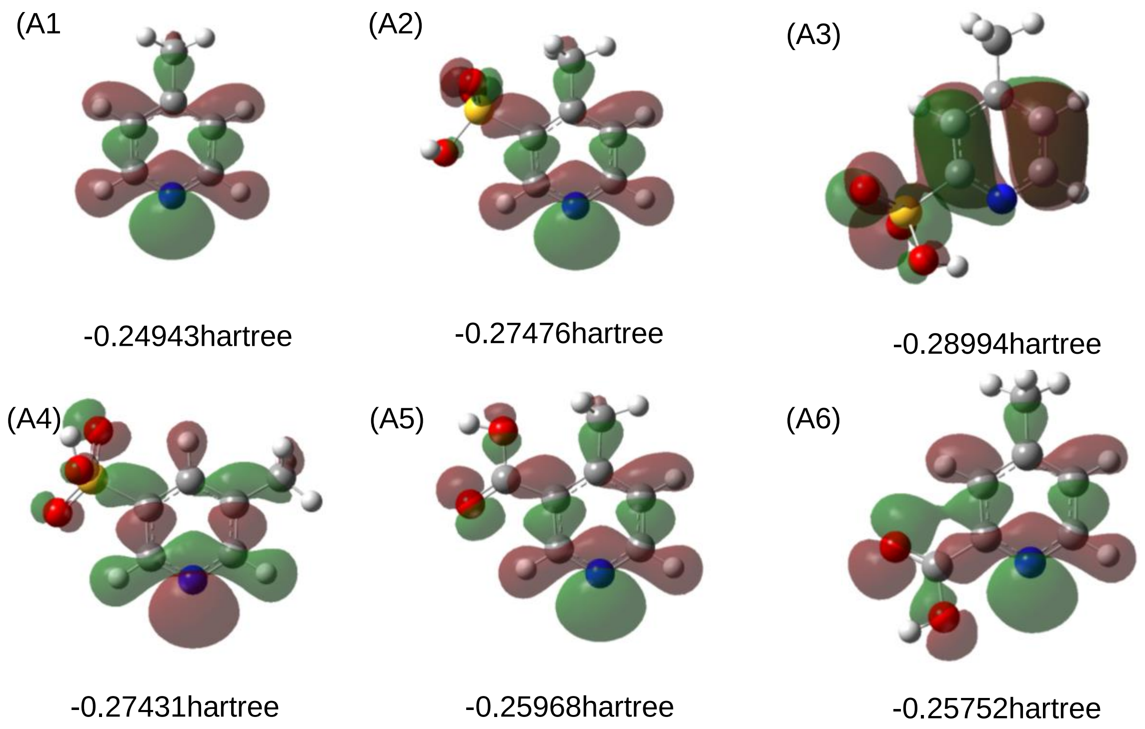

Figure S4 HOMO orbitals of six raw materials.

Compare the homo orbitals of the materials, it is easy to find that the five materials (A2,A3,A4,A5 and A6) have similar orbitals, indicating that they have similar chemical properties.

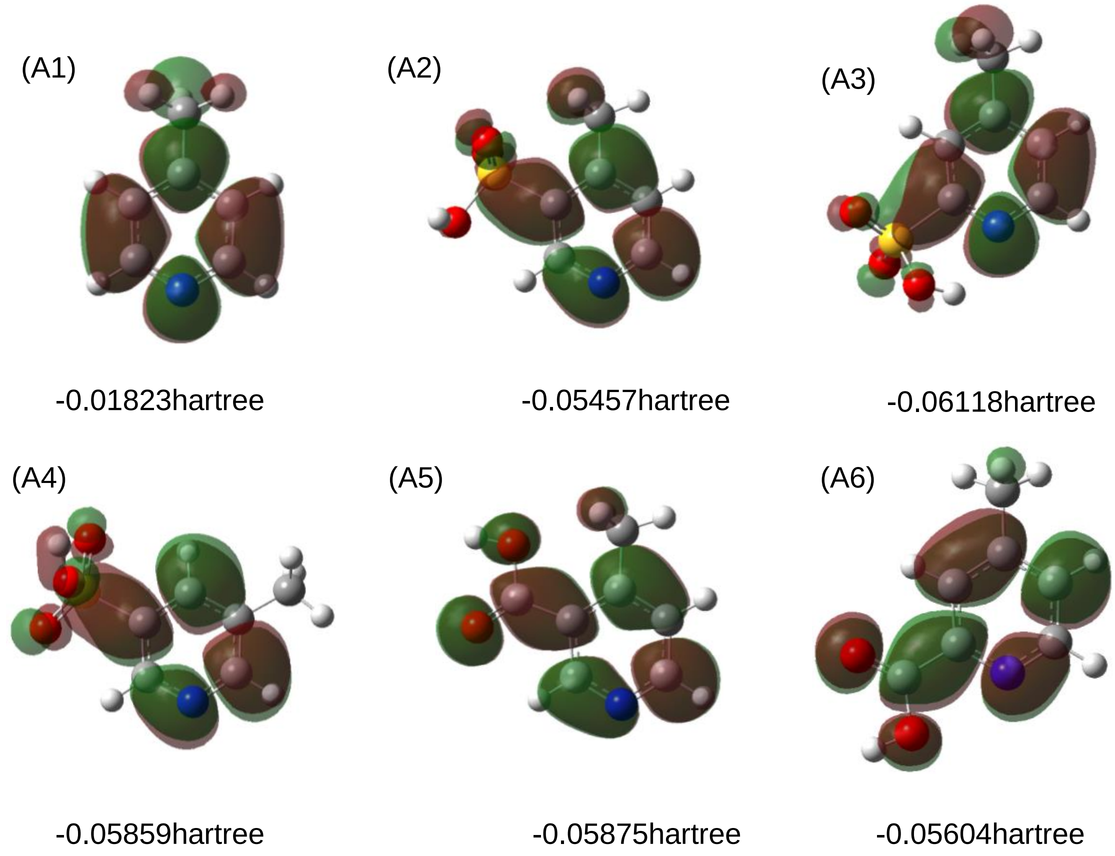

Figure S5. LUMO orbitals of six raw materials (A1, A2, A3, A4, A5, And A6).

The LUMO orbitals of A3 in Figure S5 is similar to that of A1, especially for the shape and area of orbitals of the atoms. A3 could be similar to A1 in some properties,

According to the following relationships (equations S1 and S2) between wavelength and frequency, the absorption and emission wavelengths of the N-methylacridinium chloride were calculated.

$$\lambda = \frac{C}{\nu} \qquad \text{(S1)}$$

$$\lambda_N = \frac{\nu_R}{\nu_N}\lambda_R \qquad \text{(S2)}$$

C:light speed; λ_{N}: wavelength of the light emitted or absorbed by N-methylacridinium chloride; λ_{R}: wavelength of the light emitted or absorbed by

Rhodamine B; ν_R: frequency of the light emitted or absorbed by Rhodamine B; ν_N: frequency of the light emitted or absorbed by N-methylacridinium chloride.

According to the reference [S1], frequencies of the maximum absorption and emission light of N-methylacridinium chloride in water were approximately $20\times10^{-3}cm^{-1}$ and $24\times10^{-3}cm^{-1}$. The maximum emission wavelength of Rhodamine B (in ethanol) is about 570nm, and the emission frequency in the reference [S1] was about 17.5 $x10^{-3}cm^{-1}$. So the calculated maximum absorption and emission wavelengths were 415nm and 498nm for N-methylacridinium chloride.

Table S1. Absorption wavelength, emission wavelength, and oscillator strength (f) calculated by TD-DFT method for N-methylacridinium chloride.

		**MO character**	**MO coefficient**	**Eexc (eV)**	**Wavelength**	**f**
Emission	ES1	H- > L	0.70507	2.5684	482.73	0.0984
	ES2	H- 2> L	0.44193	3.5026	353.98	0.1758
		H- 1> L	0.54299			
	ES3	H- 2> L	-0.53892	3.6221	342.30	0.3991
		H- 1> L	0.40121			
		H- > L	0.16025			
Absorption	ES1	H- > L	0.70119	3.0026	412.93	0.0633
	ES2	H- 2> L	-0.21201	3.7869	327.40	0.2432
		H- 1> L	0.62539			
		H- > L+1	-0.24883			
	ES3	H- 2> L	0.66527	3.9202	316.27	0.0236
		H- 1> L	0.17385			
		H- > L+1	-0.13750			

ES: Excited state Eexc: Energy of the excited light

Singlet oxygen ($^1O_2$*): It is kinetically unstable at ambient temperature, however the rate of decay is slow. It often appears and coexists in environment that also generates ozone, such as pine forests with photo-degradation of turpentine

Triplet oxygen ($^3O_2$): It is the most stable and common allotrope of oxygen. Molecules of triplet oxygen contain two unpaired electrons, making triplet oxygen an unusual example of a stable and commonly encountered diradical.

Superoxide(O_2^-): A superoxide is a compound that contains the superoxide anion, which has the chemical formula O_2^-. The systematic name of the anion is dioxide(-1). The reactive oxygen anion superoxide is particularly important as the product of the one-electron reduction of oxygen molecule O_2, which occurs widely in nature [S2]

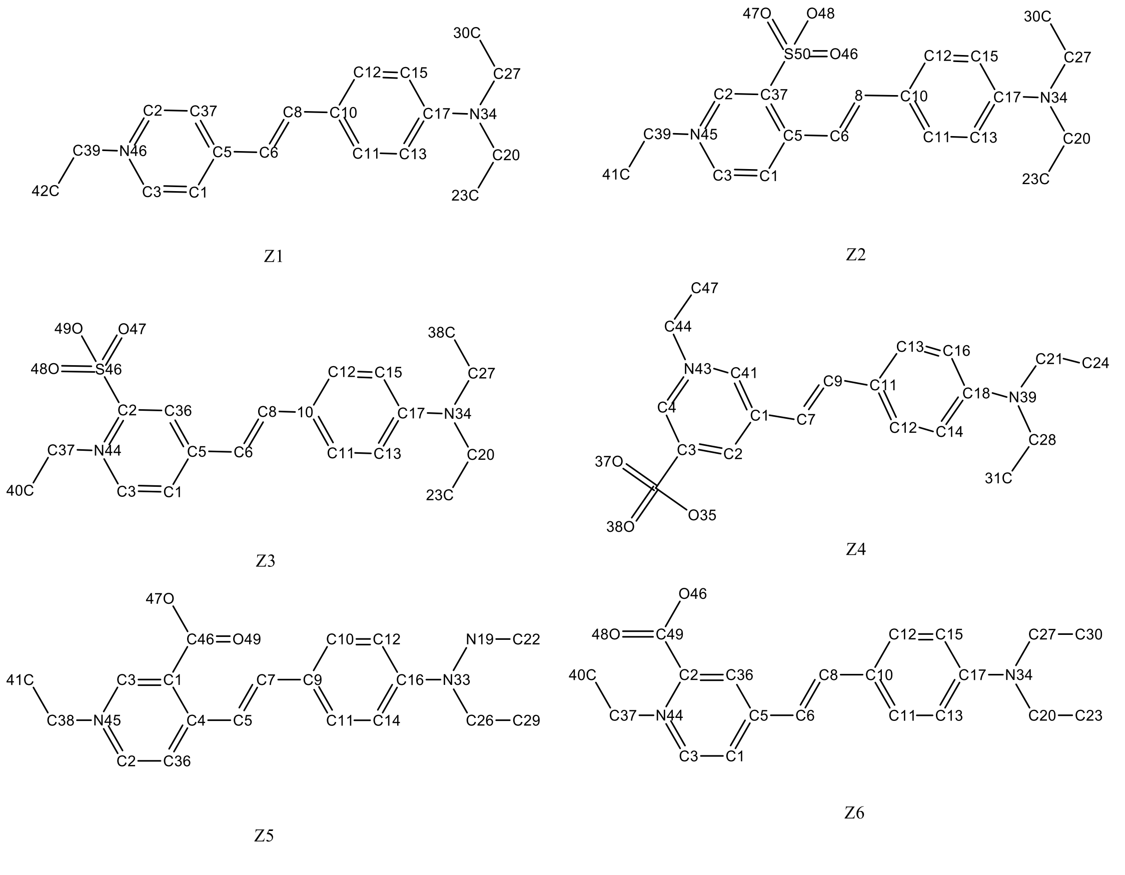

Figure S6. Numbered atoms of six dyes

Table S2. HOMO and LUMO orbitals of six designed dyes

			HOMO	LUMO				HOMO	LUMO
			atomic orbital coefficients					atomic orbital coefficients	
Z1	C1	2S	0.00029	-0.00039	Z2	C1	2S	-0.00144	-0.0013
		2PX	-0.00679	0.00517			2PX	-0.01018	0.00173
		2PY	-0.00318	0.00238			2PY	-0.00784	0.0033
		2PZ	0.09927	-0.07754			2PZ	0.10439	-0.05062
	C2	2S	-0.00022	0.00022		C2	2S	-0.00073	0.00105
		2PX	-0.00332	0.01336			2PX	-0.00483	0.01494
		2PY	-0.00037	0.00234			2PY	-0.00122	0.00636
		2PZ	0.0481	-0.18852			2PZ	0.0353	-0.14321
	C3	2S	-0.00017	0.00002		C3	2S	0.00011	0.00147
		2PX	-0.00267	0.01078			2PX	-0.00519	0.0171
		2PY	-0.00236	0.00812			2PY	-0.0045	0.01293
		2PZ	0.04272	-0.15797			2PZ	0.0669	-0.17116
	C5	2S	0.00007	-0.00034		C5	2S	0.00076	-0.00148
		2PX	0.00259	-0.0183			2PX	0.00684	-0.02186
		2PY	0.00129	-0.00852			2PY	0.00516	-0.01471
		2PZ	-0.04244	0.28803			2PZ	-0.07709	0.2952
	C6	2S	-0.00002	0.00009		C6	2S	0.00018	-0.00089
		2PX	0.01542	-0.00308			2PX	0.01609	-0.00115
		2PY	0.00645	-0.00131			2PY	0.00061	0.00048
		2PZ	-0.22738	0.03667			2PZ	-0.22962	0.00795
	C8	2S	-0.00043	-0.00037		C8	2S	-0.00028	-0.00105
		2PX	0.00084	0.01839			2PX	-0.00202	0.02014
		2PY	0.00048	0.00775			2PY	-0.00059	-0.00427
		2PZ	-0.0206	-0.26413			2PZ	0.00737	-0.27537
	C10	2S	0.0008	0.00043		C10	2S	0.00046	0.00082
		2PX	-0.01887	-0.00095			2PX	-0.01688	-0.00354
		2PY	-0.00764	-0.00069			2PY	0.00892	0.0007

		2PZ	0.26663	0.02026			2PZ	0.26526	0.04106
	C11	2S	-0.00003	0.00034		C11	2S	-0.00039	0.00024
		2PX	-0.00544	-0.01006			2PX	-0.00456	-0.0108
		2PY	-0.00178	-0.0039			2PY	0.00101	0.00382
		2PZ	0.0579	0.12864			2PZ	0.04555	0.13809
	C12	2S	-0.00019	0.00013		C12	2S	-0.00023	-0.00108
		2PX	-0.00628	-0.01013			2PX	-0.00384	-0.00803
		2PY	-0.00154	-0.00297			2PY	0.00115	0.00541
		2PZ	0.06164	0.12627			2PZ	0.04872	0.13383
	C13	2S	0.0008	0.00067		C13	2S	0.0015	0.00113
		2PX	0.01605	0.00295			2PX	0.01536	0.00449
		2PY	0.00379	-0.00077			2PY	-0.00457	-0.00174
		2PZ	-0.18354	-0.02862			2PZ	-0.18268	-0.04108
	C15	2S	0.00132	0.00096		C15	2S	0.00067	0.00114
		2PX	0.01596	0.00364			2PX	0.01231	0.00239
		2PY	0.00512	0.00196			2PY	-0.00323	0.00018
		2PZ	-0.17037	-0.0353			2PZ	-0.16838	-0.04271
	C17	2S	-0.00187	-0.00177		C17	2S	-0.00181	-0.00192
		2PX	0.01156	0.01305			2PX	0.00893	0.01217
		2PY	0.00479	0.00449			2PY	-0.00533	-0.00513
		2PZ	-0.13943	-0.13939			2PZ	-0.1272	-0.14978
	C20	2S	0.00385	0.00109		C20	2S	0.00401	0.00156
		2PX	0.00329	-0.00003			2PX	0.00237	-0.00083
		2PY	-0.00568	-0.00387			2PY	-0.00953	-0.00438
		2PZ	-0.03529	-0.00031			2PZ	-0.0349	-0.00092
	C23	2S	0.01966	0.00732		C23	2S	0.01946	0.00851
		2PX	-0.00665	0.00088			2PX	-0.01282	-0.00123
		2PY	0.0347	0.01057			2PY	0.03704	0.0136
		2PZ	0.0553	0.01852			2PZ	0.05074	0.01964
	C27	2S	0.00401	0.00143		C27	2S	0.00354	0.001

		2PX	0.00137	-0.00177			2PX	0.00221	-0.00087
		2PY	0.00848	0.00362			2PY	0.00473	0.0045
		2PZ	-0.03546	0.00003			2PZ	-0.03628	-0.00129
	C30	2S	0.02049	0.00755		C30	2S	0.01919	0.00798
		2PX	-0.00458	0.00206			2PX	0.00262	0.00471
		2PY	-0.03885	-0.01147			2PY	-0.03262	-0.0106
		2PZ	0.05289	0.01779			2PZ	0.05505	0.02115
	N34	2S	0.00654	0.00329		N34	2S	0.00641	0.0038
		2PX	-0.02614	-0.00907			2PX	-0.02303	-0.00933
		2PY	-0.0121	-0.00442			2PY	0.01389	0.0061
		2PZ	0.27263	0.10212			2PZ	0.26495	0.11526
	C37	2S	0.0004	-0.00054		C37	2S	0.00138	-0.00105
		2PX	-0.00682	0.00429			2PX	-0.00675	0.00542
		2PY	-0.00296	0.00205			2PY	-0.00623	0.00209
		2PZ	0.10157	-0.06337			2PZ	0.10857	-0.076
	C39	2S	0.00123	0.00117		C38	2S	0.0013	0.00039
		2PX	-0.00357	0.00989			2PX	-0.00391	0.00731
		2PY	-0.00073	0.00018			2PY	-0.00241	0.00287
		2PZ	0.0167	-0.0016			2PZ	0.01763	-0.00215
	C42	2S	0.0091	-0.01819		C41	2S	0.00959	-0.01592
		2PX	0.01506	-0.0165			2PX	0.01575	-0.01488
		2PY	0.00222	-0.00165			2PY	0.00757	-0.00918
		2PZ	-0.02261	0.0408			2PZ	-0.0229	0.03489
	N46	2S	0.00181	-0.00461		N45	2S	0.00203	-0.00467
		2PX	0.00522	-0.00777			2PX	0.00998	-0.01429
		2PY	0.00325	-0.00579			2PY	0.0069	-0.01036
		2PZ	-0.11832	0.22269			2PZ	-0.12534	0.19891
	-	-	-	-		O46	2S	-0.00208	0.00512
	-	-	-	-			2PX	-0.01669	0.01715
	-	-	-	-			2PY	-0.009	0.00733

	-	-	-	-			2PZ	-0.00311	0.00354
	-	-	-	-		O47	2S	0.00015	-0.00121
	-	-	-	-			2PX	0.00073	-0.00159
	-	-	-	-			2PY	0.00234	-0.00359
	-	-	-	-			2PZ	-0.01449	0.02834
	-	-	-	-		O48	2S	0.00177	-0.00377
	-	-	-	-			2PX	0.01645	-0.01598
	-	-	-	-			2PY	0.00856	-0.0062
	-	-	-	-			2PZ	0.00666	-0.00976
	-	-	-	-		S50	2S	-0.00035	0.00092
	-	-	-	-			2PX	-0.00059	0.00088
	-	-	-	-			2PY	-0.00051	0.00028
	-	-	-	-			2PZ	-0.00343	0.01022
	-	-	-	-			3S	0.00252	0.00006
	-	-	-	-			3PX	0.00167	-0.00171
	-	-	-	-			3PY	0.00143	-0.00057
	-	-	-	-			3PZ	0.01064	-0.03126
Z3			HOMO	LUMO	Z4			HOMO	LUMO
			atomic orbital coefficients					atomic orbital coefficients	
	C1	2S	-0.00044	0.00063		C1	2S	0.0003	-0.00079
		2PX	-0.00608	0.00544			2PX	0.00023	-0.00052
		2PY	0.01103	-0.00954			2PY	0.00068	-0.00883
		2PZ	0.11115	-0.09606			2PZ	0.0029	-0.15157
	C2	2S	-0.00064	0.00433		C2	2S	0.0005	0.0011
		2PX	-0.00401	0.01741			2PX	0.00098	0.00351
		2PY	0.00256	-0.0107			2PY	0.00623	0.01942
		2PZ	0.05343	-0.20734			2PZ	0.09314	0.30619
	C3	2S	-0.00007	0.00162		C3	2S	-0.00048	-0.00069
		2PX	-0.00304	0.01007			2PX	-0.00111	-0.00301
		2PY	0.00573	-0.01537			2PY	-0.00019	-0.00293

		2PZ	0.05356	-0.15098			2PZ	0.00464	-0.04722
	C5	2S	-0.00016	0.00161		C4	2S	0.00003	-0.0028
		2PX	0.00317	-0.01456			2PX	0.00212	0.00181
		2PY	-0.00567	0.02829			2PY	-0.00504	-0.0105
		2PZ	-0.05767	0.28054			2PZ	-0.10342	-0.25597
	C6	2S	-0.00011	0.00023		C7	2S	0.00025	0.00142
		2PX	0.01188	-0.00036			2PX	0.00133	0.00245
		2PY	-0.0234	0.00095			2PY	-0.01496	-0.0024
		2PZ	-0.22808	0.00457			2PZ	-0.22477	-0.02892
	C8	2S	0.00035	-0.00027		C9	2S	0.00052	0.00004
		2PX	-0.00056	0.01242			2PX	-0.00012	-0.0009
		2PY	-0.00024	-0.02674			2PY	-0.00271	0.00847
		2PZ	-0.0041	-0.25353			2PZ	-0.04143	0.13298
	C10	2S	-0.00065	-0.00028		C11	2S	-0.00034	0.00011
		2PX	-0.01275	-0.0016			2PX	-0.0019	0.00019
		2PY	0.02765	0.00423			2PY	0.01762	-0.00097
		2PZ	0.26437	0.0397			2PZ	0.26784	-0.01325
	C11	2S	0.00007	-0.00012		C12	2S	-0.00003	0.00022
		2PX	-0.00346	-0.0066			2PX	-0.00203	0.00076
		2PY	0.00541	0.01334			2PY	0.00438	-0.00428
		2PZ	0.04906	0.12396			2PZ	0.06437	-0.06239
	C12	2S	0.00034	-0.00002		C13	2S	0.00027	0.00012
		2PX	-0.00427	-0.00697			2PX	-0.00253	0.00106
		2PY	0.00547	0.01204			2PY	0.00374	-0.00337
		2PZ	0.05309	0.12103			2PZ	0.07402	-0.06312
	C13	2S	-0.0008	-0.00067		C14	2S	0.00007	0.00007
		2PX	0.01137	0.00281			2PX	0.00349	-0.00032
		2PY	-0.01804	-0.00257			2PY	-0.01067	0.00007
		2PZ	-0.18215	-0.03693			2PZ	-0.18521	0.01387
	C15	2S	-0.00136	-0.00089		C16	2S	-0.00044	0.00021

		2PX	0.01153	0.00303			2PX	0.00452	-0.00063
		2PY	-0.01809	-0.00518			2PY	-0.00772	0.00086
		2PZ	-0.1685	-0.04082			2PZ	-0.17148	0.01974
	C17	2S	0.00182	0.00169		C18	2S	0.00433	-0.00127
		2PX	0.00695	0.0088			2PX	-0.00108	-0.00128
		2PY	-0.01449	-0.01481			2PY	-0.01077	0.0046
		2PZ	-0.13001	-0.13363			2PZ	-0.14857	0.06701
	C20	2S	-0.00374	-0.00097		C21	2S	0.00288	-0.00069
		2PX	0.00327	0.00054			2PX	0.00234	-0.0005
		2PY	0.00282	0.00382			2PY	-0.00872	0.00171
		2PZ	-0.03596	-0.00159			2PZ	-0.0316	-0.00165
	C23	2S	-0.01922	-0.00733		C24	2S	0.00258	-0.00065
		2PX	-0.01022	-0.00022			2PX	0.00964	-0.00134
		2PY	-0.02897	-0.00951			2PY	0.00182	-0.00009
		2PZ	0.05683	0.01951			2PZ	0.00182	0.00128
	C27	2S	-0.00396	-0.00137		C28	2S	0.00015	-0.00081
		2PX	-0.00037	-0.00203			2PX	-0.00039	-0.00027
		2PY	-0.01093	-0.00348			2PY	0.00959	-0.0034
		2PZ	-0.03509	-0.00076			2PZ	-0.04023	0.00139
	C30	2S	-0.0199	-0.00753		C31	2S	-0.02056	0.00347
		2PX	0.00187	0.00391			2PX	0.0014	-0.00167
		2PY	0.04192	0.0126			2PY	-0.03005	0.00396
		2PZ	0.04931	0.01712			2PZ	0.06449	-0.00995
	N34	2S	-0.00645	-0.00336		O35	2S	0.00037	-0.00796
		2PX	-0.0176	-0.00622			2PX	-0.00429	-0.0103
		2PY	0.03289	0.0126			2PY	-0.00113	-0.01077
		2PZ	0.26631	0.10354			2PZ	0.00061	-0.00576
	C36	2S	-0.00034	0.00061		O37	2S	0.00158	0.0114
		2PX	-0.00601	0.00144			2PX	-0.00122	-0.01341
		2PY	0.0086	-0.00053			2PY	0.00326	0.0124

		2PZ	0.09429	-0.00005			2PZ	0.00265	0.01909
	C37	2S	-0.00119	-0.00133		O38	2S	-0.00174	-0.00734
		2PX	-0.0034	0.00891			2PX	0.00179	0.00759
		2PY	0.00342	-0.00526			2PY	-0.00334	-0.00001
		2PZ	0.01983	-0.00779			2PZ	-0.00328	-0.00603
	C40	2S	-0.00976	0.01726		N39	2S	-0.01211	0.00245
		2PX	0.01399	-0.01417			2PX	0.00047	-0.00043
		2PY	-0.01225	0.01779			2PY	0.02555	-0.00456
		2PZ	-0.0236	0.03824			2PZ	0.28064	-0.04922
	N44	2S	-0.0018	0.00306		S40	2S	0.00002	-0.00071
		2PX	0.00437	-0.00542			2PX	-0.00018	-0.00201
		2PY	-0.01105	0.01605			2PY	-0.0002	-0.00191
		2PZ	-0.13116	0.22645			2PZ	-0.00012	0.00577
	S46	2S	-0.00009	0.00092			3S	-0.00054	0.00442
		2PX	0.00098	-0.00853			3PX	0.0007	0.00764
		2PY	0	0.00085			3PY	0.00075	0.00681
		2PZ	-0.0018	0.01911			3PZ	0.00048	-0.01717
		3S	0.00097	-0.00632		C41	2S	-0.00059	-0.00072
		3PX	-0.00268	0.02476			2PX	-0.00112	0.00463
		3PY	-0.00001	-0.00174			2PY	0.00706	-0.01301
		3PZ	0.00566	-0.05756			2PZ	0.1091	-0.1681
	O47	2S	-0.00015	0.00336		N43	2S	0.00013	-0.00454
		2PX	0.00079	-0.00421			2PX	0.00035	-0.00689
		2PY	-0.00093	0.0001			2PY	-0.00106	0.00973
		2PZ	-0.01308	0.04281			2PZ	-0.01331	0.2869
	O48	2S	0.00031	0.00567		C44	2S	-0.00008	-0.00008
		2PX	0.01063	-0.05269			2PX	-0.00323	-0.00816
		2PY	0.00428	-0.01402			2PY	0.00283	-0.00782
		2PZ	-0.00063	-0.00061			2PZ	0.00197	-0.00307
	O49	2S	-0.00022	-0.00969		C47	2S	0.00129	-0.02178

		2PX	-0.00918	0.04588			2PX	0.00529	0.01557
		2PY	-0.00263	0.0103			2PY	-0.00569	0.01285
		2PZ	0.00003	-0.00881			2PZ	-0.00211	0.05113
Z5			HOMO	LUMO	Z6			HOMO	LUMO
			atomic orbital coefficients					atomic orbital coefficients	
	C1	2S	0.00125	0.00116		C1	2S	-0.00011	0.0003
		2PX	0.01566	-0.01204			2PX	-0.00507	0.00448
		2PY	0.00792	-0.00624			2PY	0.01124	-0.01043
		2PZ	0.1087	-0.09988			2PZ	0.10645	-0.09998
	C2	2S	-0.00025	-0.00281		C2	2S	-0.00064	0.00492
		2PX	0.01002	-0.03309			2PX	0.00218	0.00306
		2PY	-0.00043	0.00089			2PY	0.00687	-0.02172
		2PZ	0.07165	-0.20217			2PZ	0.04576	-0.20415
	C3	2S	0.00159	-0.00809		C3	2S	0.00006	-0.00115
		2PX	0.00623	-0.02267			2PX	-0.00117	0.00211
		2PY	-0.00122	0.00051			2PY	0.00738	-0.02243
		2PZ	0.02762	-0.09251			2PZ	0.04999	-0.14513
	C4	2S	-0.00158	0.00428		C5	2S	0.00016	-0.00135
		2PX	-0.00772	0.02464			2PX	0.00233	-0.01331
		2PY	-0.00351	0.0017			2PY	-0.005	0.02768
		2PZ	-0.07076	0.29174			2PZ	-0.05294	0.2769
	C5	2S	0.00094	0.00291		C6	2S	0.0005	-0.00135
		2PX	-0.009	-0.00229			2PX	0.0141	-0.00275
		2PY	0.01555	0.00059			2PY	-0.02161	0.00162
		2PZ	-0.22915	0.00028			2PZ	-0.22728	0.01048
	C7	2S	0.00179	-0.00038		C8	2S	0.0007	0.00142
		2PX	-0.00184	-0.00435			2PX	0.00023	0.01683
		2PY	0.0006	0.01598			2PY	-0.0002	-0.02262
		2PZ	0.00259	-0.27098			2PZ	-0.00634	-0.24951
	C9	2S	-0.0012	0.00103		C10	2S	-0.00092	-0.00086

		2PX	0.00995	-0.00204			2PX	-0.01844	-0.00239
		2PY	-0.0119	-0.0017			2PY	0.02312	0.00327
		2PZ	0.26466	0.03411			2PZ	0.26547	0.03563
	C10	2S	-0.00017	-0.00154		C11	2S	0.00008	-0.00061
		2PX	0.00396	0.00807			2PX	-0.00493	-0.00963
		2PY	-0.00161	-0.00518			2PY	0.00453	0.01078
		2PZ	0.05024	0.13384			2PZ	0.0501	0.12148
	C11	2S	0.00072	-0.00016		C12	2S	0.00014	-0.00012
		2PX	0.00379	0.00663			2PX	-0.00564	-0.0093
		2PY	-0.0024	-0.00462			2PY	0.00485	0.0097
		2PZ	0.05482	0.13238			2PZ	0.05428	0.11893
	C12	2S	-0.00049	0		C13	2S	-0.0007	-0.00049
		2PX	-0.01124	-0.00329			2PX	0.01625	0.0035
		2PY	0.00632	-0.00024			2PY	-0.01474	-0.00166
		2PZ	-0.18032	-0.03314			2PZ	-0.18321	-0.03443
	C14	2S	-0.0013	-0.00065		C15	2S	-0.00121	-0.00091
		2PX	-0.01098	-0.00314			2PX	0.01567	0.00365
		2PY	0.00721	0.00266			2PY	-0.01516	-0.00424
		2PZ	-0.1681	-0.04197			2PZ	-0.16987	-0.03937
	C16	2S	0.00205	0.00185		C17	2S	0.00187	0.00168
		2PX	-0.00779	-0.00997			2PX	0.01073	0.01212
		2PY	0.00668	0.00703			2PY	-0.01215	-0.01194
		2PZ	-0.13186	-0.14634			2PZ	-0.1317	-0.13091
	C19	2S	-0.00385	-0.0012		C20	2S	-0.0039	-0.00106
		2PX	-0.00245	-0.00007			2PX	0.00379	0.00028
		2PY	-0.00478	-0.0039			2PY	0.0035	0.00364
		2PZ	-0.03478	-0.00088			2PZ	-0.03613	-0.00132
	C22	2S	-0.01898	-0.0078		C23	2S	-0.01942	-0.00713
		2PX	0.00594	-0.00112			2PX	-0.0098	-0.00011
		2PY	0.03282	0.01118			2PY	-0.03104	-0.00964

		2PZ	0.0541	0.01996			2PZ	0.05656	0.01873
	C26	2S	-0.00402	-0.00157		C27	2S	-0.00403	-0.00134
		2PX	-0.00016	0.00208			2PX	0.00091	-0.00186
		2PY	0.00865	0.00373			2PY	-0.01032	-0.00344
		2PZ	-0.0345	-0.00018			2PZ	-0.03562	-0.00069
	C29	2S	-0.01981	-0.0081		C30	2S	-0.02022	-0.00739
		2PX	0.00257	-0.0029			2PX	-0.00172	0.00276
		2PY	-0.03848	-0.01257			2PY	0.04152	0.0121
		2PZ	0.05065	0.01878			2PZ	0.05054	0.01691
	N33	2S	-0.00608	-0.00341		N34	2S	-0.00637	-0.00326
		2PX	0.01877	0.00698			2PX	-0.02539	-0.00889
		2PY	-0.01588	-0.00643			2PY	0.02755	0.01014
		2PZ	0.26451	0.10986			2PZ	0.26918	0.10063
	C36	2S	0.0034	-0.00284		C36	2S	-0.00105	-0.00025
		2PX	0.0166	-0.0032			2PX	-0.00492	-0.00531
		2PY	0.00346	-0.00243			2PY	0.01103	-0.00113
		2PZ	0.10221	-0.0229			2PZ	0.097	-0.00598
	C38	2S	-0.00125	-0.00089		C37	2S	-0.00141	0.00176
		2PX	0.00504	-0.00786			2PX	-0.00113	0.00348
		2PY	0.00109	-0.00476			2PY	0.00398	-0.0032
		2PZ	0.01686	-0.00022			2PZ	0.01937	-0.00673
	C41	2S	-0.00927	0.01412		C40	2S	-0.0094	0.01791
		2PX	-0.01684	0.01479			2PX	0.01432	-0.01658
		2PY	-0.00575	0.01108			2PY	-0.0104	0.01533
		2PZ	-0.02187	0.0326			2PZ	-0.02216	0.03489
	N45	2S	-0.00167	0.00367		N44	2S	-0.00092	0.00566
		2PX	-0.0167	0.02376			2PX	0.00373	-0.00802
		2PY	0.00269	-0.00222			2PY	-0.01395	0.03262
		2PZ	-0.12247	0.18462			2PZ	-0.12471	0.22579
	C46	2S	0.0009	0.00386		O46	2S	-0.00358	0.00822

		2PX	0.0101	-0.02834			2PX	0.00571	0.0071
		2PY	0.0018	-0.00346			2PY	0.01004	-0.03877
		2PZ	0.01973	-0.05825			2PZ	-0.00865	0.04575
	O47	2S	-0.0058	0.00711		O48	2S	-0.00051	-0.00041
		2PX	-0.01141	0.01545			2PX	-0.01218	0.06689
		2PY	0.00429	-0.00121			2PY	0.00289	-0.00785
		2PZ	-0.02007	0.04323			2PZ	-0.01257	0.07017
	O49	2S	0.00007	-0.00517		C49	2S	0.00102	0.00255
		2PX	-0.02139	0.02679			2PX	0.00259	-0.04527
		2PY	-0.00177	0.01305			2PY	-0.00104	0.01124
		2PZ	-0.0292	0.05823			2PZ	0.00278	-0.06478

Table S3. Energy gaps between HOMO/LUMO orbitals of dyes and HOMO/LUMO orbitals of $^{1}O_2^{*}$/ O_2^{-}

	**Dye's HOMO (hatree)**	**$^{*1}O_2$ LUMO (hatree)**	**ΔE (hatree)**	**Dye's LUMO (hatree)**	**O_2^{-} HOMO (hatree)**	**ΔE (hatree)**
Z1	-0.35155	0.01129	0.36284	-0.08185	-0.37441	0.29256
Z2	-0.3608		0.37209	-0.0966		0.27781
Z3	-0.36091		0.3722	-0.10292		0.27149
Z4	-0.34589		0.35718	-0.08963		0.28478
Z5	-0.3542		0.36549	-0.08735		0.28706
Z6	-0.36121		0.3725	-0.10124		0.27317

Table S4. The charges of the atoms of dyes calculated by Gaussian 09

**Z1**		**Z2**		**Z3**		Z4		Z5		Z6	
**C1**	**-0.30348**	**C1**	**-0.30247**	**C1**	**-0.29855**	C1	0.0027	C1	-0.27226	C1	-0.30117
C2	0.162789	C2	0.199262	C2	0.069216	C2	-0.09137	C2	0.157865	C2	0.303921
C3	0.160028	C3	0.162809	C3	0.167607	C3	-0.3845	C3	0.218619	C3	0.165018
C5	0.167447	C5	0.218266	C5	0.17475	C4	0.171643	C4	0.227281	C5	0.177661
C6	-0.30075	C6	-0.33042	C6	-0.30492	C7	-0.28338	C5	-0.34389	C6	-0.30582
C8	-0.10686	C8	-0.09985	C8	-0.10081	C9	-0.13187	C7	-0.08882	C8	-0.10255
C10	-0.05219	C10	-0.06273	C10	-0.05596	C11	-0.0424	C9	-0.06542	C10	-0.05512
C11	-0.16618	C11	-0.15675	C11	-0.1605	C12	-0.17599	C10	-0.15823	C11	-0.1619
C12	-0.1558	C12	-0.15154	C12	-0.15181	C13	-0.16265	C11	-0.15013	C12	-0.15252
C13	-0.29244	C13	-0.29437	C13	-0.29277	C14	-0.28822	C12	-0.29531	C13	-0.29261
C15	-0.28842	C15	-0.29088	C15	-0.28843	C16	-0.29201	C14	-0.28997	C15	-0.28802
C17	0.462705	C17	0.471485	C17	0.46835	C18	0.45528	C16	0.468881	C17	0.467238
C20	-0.13492	C20	-0.13897	C20	-0.13801	C21	-0.09896	C19	-0.13684	C20	-0.13767
C23	-0.4864	C23	-0.48697	C23	-0.48727	C24	-0.52091	C22	-0.48697	C23	-0.48723
C27	-0.13493	C27	-0.1388	C27	-0.13817	C28	-0.14069	C26	-0.13649	C27	-0.13761
C30	-0.4857	C30	-0.48815	C30	-0.48707	C31	-0.48557	C29	-0.4861	C30	-0.48653
N34	-0.71421	N34	-0.70994	N34	-0.71106	O35	-0.77609	N33	-0.71222	N34	-0.71178

C37	-0.31255	C37	-0.4697	C36	-0.29361	O37	-0.62992	C36	-0.33051	C36	-0.32834
C39	-0.16809	C38	-0.16926	C37	-0.20142	O38	-0.63829	C38	-0.16685	C37	-0.16367
C42	-0.488	C41	-0.48984	C40	-0.49221	N39	-0.72386	C41	-0.48991	C40	-0.49443
N46	-0.63095	N45	-0.64881	N44	-0.66154	S40	1.696887	N45	-0.65182	N44	-0.70271
-	-	O46	-0.64219	S46	1.736886	C41	0.133202	C46	0.836789	O46	-0.68429
-	-	O47	-0.66781	O47	-0.63741	N43	-0.58931	O47	-0.7299	O48	-0.49842
-	-	O48	-0.7962	O48	-0.62242	C44	-0.18173	O49	-0.57422	C49	0.814309
-	-	S50	1.7498	O49	-0.79432	C47	-0.49636	-	-	-	-

The charges of the hydrogen atoms are not listed in Table S4 because that all the hydrogen atoms have positive charges, the atoms of dyes are numbered in Figure S6. From Table S4 and Figure S6, it easy to notice that the carbon atoms in the bridge bonds have negative charges, while the most atoms in Ph rings have negative charges but half atoms in Py rings have positive charges. Although, N is the most electronegative atom in dyes Z1 and Z6, different to the Z1, the carbon in carboxyl has the most positive charge in dye Z6, while the most electronegative atoms of rest four dyes are oxygen atom. The sulfur atoms in dyes Z2-Z4 and the carbon atom in carboxyl group of dye Z5 have the most positive charge. It meant that the N in dyes Z1 and Z6 were more vulnerable than N atom in dyes Z2-Z5 since the O were more negative, which could be illustrated as the sulfonic group and carboxyl group in dyes Z2-Z5 could protect the N atoms. In other words, the dyes Z2-Z5 are still can emit fluorescence even the sulfonic or carboxyl groups were destroyed.

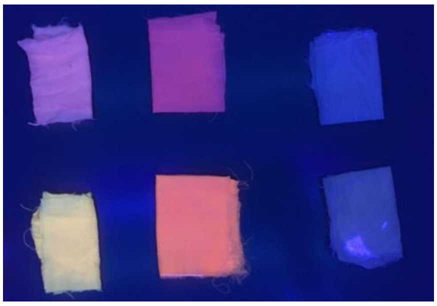

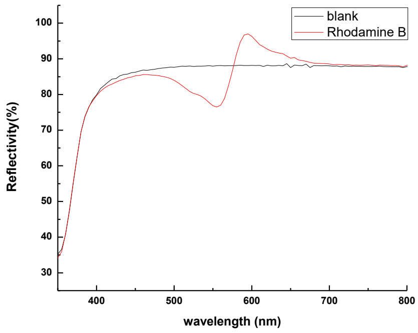

(a) (b)

Figure S7. (a) The fabrics (PAN, Nylon and cotton from left to right) dyed by dyes Z2 (upper) and Z5 (bottom) under UV light; (b) the reflectivity of PET dyed by Rhodamine B at 130°C.

The Figure S7b shown the reflectivity of the PET fiber dyed by Rhodamine b, it's easy to find the absorption and emission peak in the curve even the intensity of the peak was weak, it could be illustrate that the Rhodamine B could dye PET fiber when the dyeing-temperature was 130°C, thus, the HSP distance would be credible to predict the dyeing properties of dyes. As shown in Figure S7, Z2 and Z5 display the different color and fluorescence when they applied to color different fibers, similar to the solvent effect, the color and fluorescence of the Z2 and Z5 would be affected by the functional groups in fibers, such like the C≡N in PAN, and CO-NH in Nylon, the different group and structure of the fiber would affect the dyes differently, the influence of the fiber would be similar to the solvation effects. Not only the Z2 and Z5 display the different color and fluorescence, some commercial fluorescent dyes display the different color and fluorescence on different fibers, as shown in figure S8, Rhodamine B display the different color and fluorescence on PAN and Nylon.

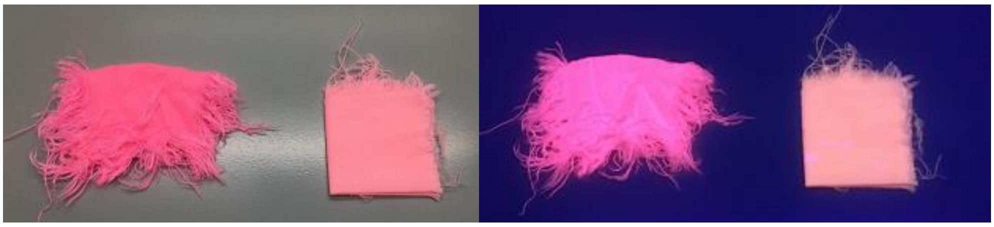

(a) (b)

Figure S8. PAN (left) and Nylon (right) dyed by Rhodamine B under D65 (a) and UV (b) light.

Table S5. The maximum absorption wavelengths, maximum excited energies, oscillator strengths, and MO characters of theoretical calculation in water based on different methods for dye Z5

Item	Wavelength (nm)	Energy (eV)	Oscillator strength (f)	MO character
b3lyp/6-311+G(d,p)	518.06	2.3932	1.2232	H→L
b3lyp/6-31+G(d,p)	518.64	2.3906	1.2194	H→L
b3lyp/6-311G(d)	509.6	2.4330	1.2638	H→L
mpw1pw91/6-311+G(d,p)	505.60	2.4522	1.2842	H→L
mpw1pw91/6-311+G(d)	504.16	2.4592	1.2870	H→L
camb3lyp/6-311+G(d,p)	468.46	2.6466	1.3895	H-1→L<br>H→L
b3pw91/6-311+G(d,p)	517.17	2.3974	1.2325	H→L
hseh1pbe/6-311+G(d,p)	515.12	2.4069	1.2451	H→L

As shown in Table S5, the different methods would give different results in varied error ranges. Compared with the experiment data in Figure S7, program mpw1pw91/6-311+G(d) produced better estimated results.

Table S6. The maximum absorption wavelengths of Z5 based on Gaussian 09 at mpw1pw91/6-311+G(d) level in water.

Item	Wavelength (nm)	Energy (eV)	Oscillator strength (f)	MO character	MO coefficient
Excited state 1	504.16	2.4592	1.2870	H→L	0.70511
Excited state 2	384.51	3.2244	0.0955	H→L+1	0.69778
Excited state 3	314.20	3.9460	0.0094	H→2-L<br>H→L+2<br>H→L+3	0.67570<br>0.11766<br>-0.14263

Table S7. The maximum emission wavelengths of Z5 based on Gaussian at b3lyp/6-31+G(d,p) level in water.

Item	Wavelength (nm)	Energy (eV)	Oscillator strength (f)	MO character	MO coefficient
Excited state 1	618.27	2.0053	1.2191	H→L	0.70474
Excited state 2	439.03	2.8240	0.1823	H→L+1	0.70239
Excited state 3	343.34	3.6111	0.0502	H-1→L<br>H→L+2	-0.67612<br>-0.16554

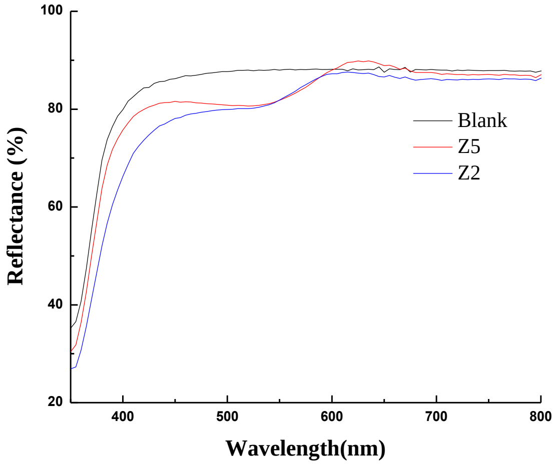

Figure S9. (a)Reflectance of the PET dyed by Z2 and Z5 at 130°C

As shown in Figure S9, Z2 and Z5 dyeing PET at 130°C successfully-have obvious absorption an emission wavelength. dyeing properties of the fibers.

^{1}HNMR of Z2 and Z5; Z2: ^{1}H NMR (600 MHz, D_2o) δ: 9.16 (Py-H), 8.81 (Py-H), 8.53 (Ph-H), 8.02(-CH=CH-), 7.46(Ph-H), 4.66($-CH_2-$), 2.89($-CH_2-$), 1.65($-CH_3$), 1.19($-CH_3$). Z5: ^{1}H NMR (600 MHz, D_2o) δ: 8.65(Py-H), 8.44(Py-H), 8.18(Py-H), 7.78(Ph-H), 7.66(-CH=CH-), 6.92(Ph-H), 4.49($-CH_2-$), 3.47($-CH_2-$), 1.62($-CH_3$), 1.19($-CH_3$).

Dyeing experiment

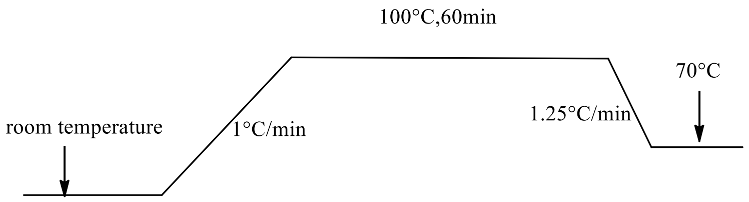

Figure S10. Time-temperature profile of dyeing process

The synthesized dyes and commercial dye Rhodamine B were used to dye PAN, celloluse and nylon following the Figure S10. The fabrics were dyed in an X-5 DYEING machine (Foshan HUANGJU, China), and the dye solutions were prepared with the required amount of each dye, sodium sulphate (3g/L) and a surfactant

(0.5g/L). The pH of the dye bath was maintained at 4.5-5.0 by acetic acid-sodium acetate buffer solution. The liquor-to-goods ratio was kept at 50:1. After immersing the fabrics into the dye solutions at room temperature, the temperature was increased to 100℃ at the rate of 1℃/min and maintained at the temperature for 60 minutes, the dye solution was cooled to 70℃ at 1.25℃/min. At the end of the dyeing, the dyed fabric was rinsed thoroughly in distilled water and allowed to dry in the open air.

The synthesized dyes and commercial dye Rhodamine B were used to dye PET fabric following the Figure S11, The PET fabrics were dyed in an X-5 DYEING machine (Foshan HUANGJU, China), and the dye solutions were prepared with the required amount of each dye, and a dispersant NNO (0.5g/L). The liquor-to-goods ratio was kept at 50:1. After immersing the PET fabric into the dye solutions at room temperature, the temperature was increased to 130℃ at the rate of 2℃/min and maintained at the temperature for 60 minutes, the dye solution was cooled to 70℃ at 3℃/min. At the end of the dyeing, the dyed fabric was rinsed thoroughly in distilled water and allowed to dry in the open air.

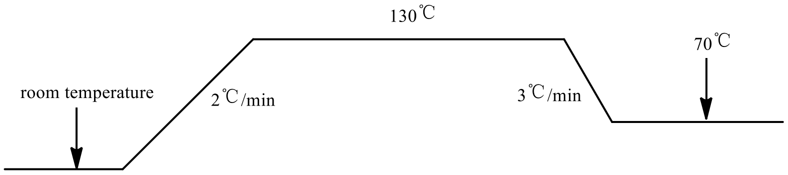

Figure S11. Dyeing process of PET fabric

References

S1. S.J. Strickler, R.A. Berg, *J Chem Phys,* 1962, **3**, 814-822

S2. M. Hayyan, M.A. Hashim, I.M. AlNashef, *Chem Rev*, 2016, **116**, 3029-3085.
